# Ca^2+^/Calmodulin-dependent protein Kinase II interacts with group I Metabotropic Glutamate and facilitates Receptor Endocytosis and ERK1/2 signaling: role of β-Amyloid

**DOI:** 10.1186/s13041-015-0111-4

**Published:** 2015-03-26

**Authors:** Fitore Raka, Andrea R Di Sebastiano, Stephanie C Kulhawy, Fabiola M Ribeiro, Christina M Godin, Fabiana A Caetano, Stephane Angers, Stephen S G Ferguson

**Affiliations:** J. Allyn Taylor Centre for Cell Biology, Robarts Research Institute, and the Department of Physiology and Pharmacology, University of Western Ontario, 100 Perth Dr. London, Ontario, N6A 5K8 Canada; Departamento de Bioquimica e Imunologia, ICB, Universidade Federal de Minas Gerais, Belo Horizonte, 31270-901 Brazil; Leslie Dan Faculty of Pharmacy, University of Toronto, Room 901 144 College Street, Toronto, Ontario Canada

**Keywords:** G protein-coupled receptors, Metabotropic Glutamate Receptors, Ca2+/Calmodulin-dependent protein Kinase IIα, Receptor Signaling, Amyloid-β

## Abstract

**Background:**

Agonist stimulation of Group I metabotropic glutamate receptors (mGluRs) initiates their coupling to the heterotrimeric G protein, Gα_q/11_, resulting in the activation of phospholipase C, the release of Ca^2+^ from intracellular stores and the subsequent activation of protein kinase C. However, it is now recognized that mGluR5a also functions as a receptor for cellular prion protein (PrP^C^) and β-amyloid peptide (Aβ42) oligomers to facilitate intracellular signaling via the resulting protein complex. Intracellular mGluR5a signaling is also regulated by its association with a wide variety of intracellular regulation proteins.

**Results:**

In the present study, we utilized mass spectroscopy to identify calmodulin kinase IIα (CaMKIIα) as a protein that interacts with the second intracellular loop domain of mGluR5. We show that CaMKIIα interacts with both mGluR1a and mGluR5a in an agonist-independent manner and is co-immunoprecipitated with mGluR5a from hippocampal mouse brain. CaMKIIα positively regulates both mGluR1a and mGluR5a endocytosis, but selectively attenuates mGluR5a but not mGluR1a-stimulated ERK1/2 phosphorylation in a kinase activity-dependent manner. We also find that Aβ42 oligomers stimulate the association of CaMKIIα with mGluR5a and activate ERK1/2 in an mGluR5a-dependent manner. However, Aβ42 oligomer-stimulated ERK1/2 phosphorylation is not regulated by mGluR5a/CaMKIIα interactions suggesting that agonist and Aβ42 oligomers stabilize distinct mGluR5a activation states that are differentially regulated by CaMKIIα. The expression of both mGluR5a and PrP^C^ together, but not alone resulted in the agonist-stimulated subcellular distribution of CaMKIIα into cytoplasmic puncta.

**Conclusions:**

Taken together these results indicate that CaMKIIα selectively regulates mGluR1a and mGluR5a ERK1/2 signaling. As mGluR5 and CaMKIIα are involved in learning and memory and Aβ and mGluR5 are implicated in Alzheimer’s disease, results of these studies could provide insight into potential pharmacological targets for treatment of Alzheimer’s disease.

## Introduction

G protein-coupled receptors (GPCRs) comprise the largest family of transmembrane receptors and function to transduce extracellular signals into intracellular responses [1,2]. GPCRs are composed of seven transmembrane domains that can be activated by a variety of stimuli including photons, odorants, hormones, amino acids, peptides and neurotransmitters [2]. Glutamate, the major excitatory neurotransmitter in the central nervous system, signals via the activation of both metabotropic GPCRs and ionotropic receptors [3-6]. There are eight different GPCRs that respond to glutamate and mediate the metabotropic glutamate receptor signaling in the brain [5-7]. These receptors are classified into three subgroups based on sequence similarity and G protein coupling specificity. Group I metabotropic glutamate receptors, (mGluR1 and mGluR5), regulate excitatory synaptic signaling response via their coupling to the heterotrimeric G protein, Gα_q/11_, which upon activation stimulates the activity of the enzyme phospholipase C to generate the production of two second messengers, diacylglycerol and inositol 1, 4, 5 trisphosphate. Inositol 1, 4, 5 trisphosphate acts as a ligand that binds to inositol phosphate receptors localized to the endoplasmic reticular membrane to release intracellular Ca^2+^ stores, in turn Ca^2+^ functions in combination with diacylglycerol to activate protein kinase C [3].

Group I mGluRs serve as molecular scaffold proteins to regulate both extracellular and intracellular signaling complexes. Specifically, mGluR5 functions as a receptor to mediate intracellular Ca^2+^ signaling and extracellular-regulated protein kinase (ERK1/2) activation by cellular prion protein (PrP^c^) and β42-amyloid (Aβ42) oligomers [8,9]. PrP^c^ can physically associate with mGluR5, and binding of Aβ to PrP^C^ generates mGluR5-mediated increases in intracellular Ca^2+^ concentrations in neurons [9]. Negative allosteric modulators of mGluR5 weaken the interaction of PrP^c^ with mGluR5, while positive allosteric modulators increase this interaction. Silent allosteric modulators of mGluR5 disrupt Aβ induced interactions of mGluR5 with PrP^c^ [10], confirming the physical interaction of these proteins and suggesting a mechanism by which PrP^c^ and Aβ can regulate mGluR5 signaling. It is now recognized that mGluR5 activation by both PrP^c^ and Aβ42 oligomers also plays an important role in the pathophysiology associated with Alzheimer’s disease (AD) [9,11]. Consistent with this concept, mGluR5 selective antagonists improve the cognitive deficits observed in AD mouse models and the genetic deletion of mGluR5 results in a reduction in both soluble Aβ42 oligomers and β-amyloid plaques and results in the amelioration of cognitive dysfunction observed in APPswePSIΔE9 AD transgenic mice [11].

Group I mGluRs, in addition to their coupling to Gα_q/11_ heterotrimeric G proteins, also interact with and scaffold a variety of intracellular signaling proteins that play a pivotal role in regulating mGluR1/5 signaling, endocytosis, subcellular localization and synaptic activity [3,12]. These proteins include, but are not limited to, Homer, calmodulin, SIAH-1a, β-arrestin1, casein kinase 1, protein phosphatase 2a, phospholipase D2, RalA, Pyk2, and Fyn [9,13-22]. β-Arrestin1 has been shown to be involved in mGluR1-mediated ERK1/2 phosphorylation, as have Pyk2, PKC and Src [22,23]. Phospholipase D2 and RalA regulate the constitutive endocytosis of Group I mGluRs, as well as a number of other GPCRs [17,24,25]. In addition, Homer interactions with Group I mGluRs regulates their G protein signaling, coupling to the activation of ERK1/2 and plays a role in Group I mGluR-mediated regulation of synaptic activity [26-28]. Both mGluR1a and mGluR5a encode a protein phosphatase 1γ binding motif and protein phosphatase 2A has been implicated in N-methyl-D-aspartate (NMDA) receptor-stimulated increases in mGluR5 activity by promoting the dephosphorylation of desensitized receptors [21,29].

Emerging studies reveal that Ca^2+^/calmodulin-dependent protein kinase IIα (CaMKIIα) can also regulate several GPCRs. CaMKIIα has been shown to regulate behavioral responses to cocaine by regulating the D3 dopamine receptor by binding to third intracellular loop of the receptor to mediate its desensitization [30]. CaMKIIα-dependent desensitization has also been reported for the D1/D2 heterodimer [31] and the Histamine H1 receptor [32]. In contrast, CaMKIIα binding to the second intracellular loops of D1/D2 receptor heteromers increases signaling [33]. Moreover, antagonism of CaMKIIα activity has been shown to reduce mGluR1 internalization [34,35]. CaMKIIα has been shown to play a direct role in regulating the desensitization of mGluR1a inositol 1, 4, 5 trisphosphate signaling by physically interacting with the C-terminal tail of the receptor [36]. Recently, CaMKIIα has also been shown to interact with the proximal C-terminal tail of mGluR5 *in vitro* [37]. In rat striatal neurons, inactive CaMKIIα binds constitutively to mGluR5 and mGluR5- mediated Ca2+ release results in the dissociation of C-tail bound CaMKIIα and recruitment to the NMDA receptor where it can phosphorylate the GluN2B subunit [37].

In the present study, we utilized a cell permeant Tat peptide from HIV [38] coupled to a peptide encoding the second intracellular loop (IL2) of mGluR1/5 followed by a FLAG epitope tag to perform a proteomic screen to identify neuronal proteins that interact with Group I mGluRs. As a consequence, we have identified a series of known Group I-interacting proteins including: protein phosphatase 1γ, protein phosphatase 2A and Gα_q11_ in the proteomic screen, as well as CaMKIIα, β, δ, and γ as novel proteins that interact with IL2 of mGluR1/5. We find that CaMKIIα positively regulates the endocytosis of both mGluR1a and mGluR5a, but has differential effects on mGluR1/5-stimulated ERK1/2 phosphorylation. Furthermore, Aβ42 oligomers stimulate mGluR5-mediated ERK1/2 phosphorylation and CaMKIIα association with mGluR5, but CaMKIIα does not regulate Aβ42 oligomer-stimulated ERK1/2 phosphorylation. In addition, mGluR5a and PrP^C^ expression results in the subcellular redistribution of CaMKIIα. Taken together our observations indicate that CaMKII regulates agonist-stimulated Group I mGluR signaling, but not Aβ42 oligomer-mediated signaling via mGluR5a.

## Results

### Identification of novel Group I-interacting proteins by mass spectroscopy

In order to identify potentially novel mGluR1/5 IL2 interacting proteins, we performed a proteomic screen using a Tat-tagged mGluR1/5 IL2 peptide conjugated to a FL-epitope tag to screen by mass spectroscopy proteins in neurons that may bind to mGluR1/5. To do this, a mixed culture of 10^7^ cortical and striatal neurons was incubated with the mGluR1/5 Tat peptide (70 μM final concentration) for 2 hours. We found that a number of novel and known interacting proteins were identified (Table 1). The known mGluR1/5 interacting proteins include Gα_q/11_, protein phosphatase 1γ catalytic subunit, and the regulatory subunit of protein phosphatase 2A [19,21]. Amongst the novel mGluR1/5 IL2-interacting proteins identified were CaMKIIα, β, δ, and γ. As CaMKII antagonism was previously reported to antagonize mGluR1a internalization [34], we examined whether CaMKIIα interacted with mGluR1a and mGluR5a to regulate either their signaling or endocytosis.

**Table 1:** **Proteins co-precipitated with Tat-mGluR1/5-IL2-FLAG peptide from mouse neuronal cultures**

**Gene ID**	**Protein names**	**Unique peptides**	**Total peptides**	**% Coverage**
12934	Dihydropyrimidinase-like 2	23	120	53
65254	Dihydropyrimidinase-like 5	11	77	29.9
15481	Heat shock protein 8	15	32	28.9
51792	α-subunit of regulatory subunit A, PP2A	8	17	21.6
12323	Ca^2+^/Calmodulin Kinase IIβ	6	41	21.6
26413	Mitogen-activated protein kinase I	4	7	19
12332	Ca^2+^/Calmodulin Kinase IIα	6	16	18.8
12325	Ca^2+^/Calmodulin Kinase IIγ	5	30	17.6
217342	Ubiquitin-conjugating enzyme E2O	16	42	17.2
22240	Dihydropyrimidinase-like 3	7	16	17.2
233726	Importin 7	16	42	17.0
12995	Casein Kinase 2 α1 polypeptide	4	8	15.6
108058	Ca^2+^/Calmodulin Kinase IIδ	4	27	15.3
69654	Dynactin 2	4	9	13.9
12331	Adeylate cyclase-associated protein 1	3	7	12.4
19047	Protein phosphatase 1γ catalytic subunit	2	6	9.6
13628	Eukaryotic elongation factor 1 alpha 1	4	8	8.2
16565	Kinesin family member 21B	5	8	6.2
13191	Dynactin 1	5	6	6.2
14682	Guanine nucleotide binding protein αq polypeptide	1	2	5.3
13175	Doublecortin-like kinase 1	3	5	5.2
67300	Clathrin heavy chain	5	6	4.1
8120	Adaptor-related protein complex 3, beta 2 subunit	3	6	3.2
69116	Ubiquitin protein ligase E3 component N-recognin 4	7	11	2.8
22215	Homologous to the E6-AP (UBE3A)	7	9	2.2

### Co-immunoprecipitation of CaMKIIα with mGluR1a and mGluR5a

**Figure 1:**
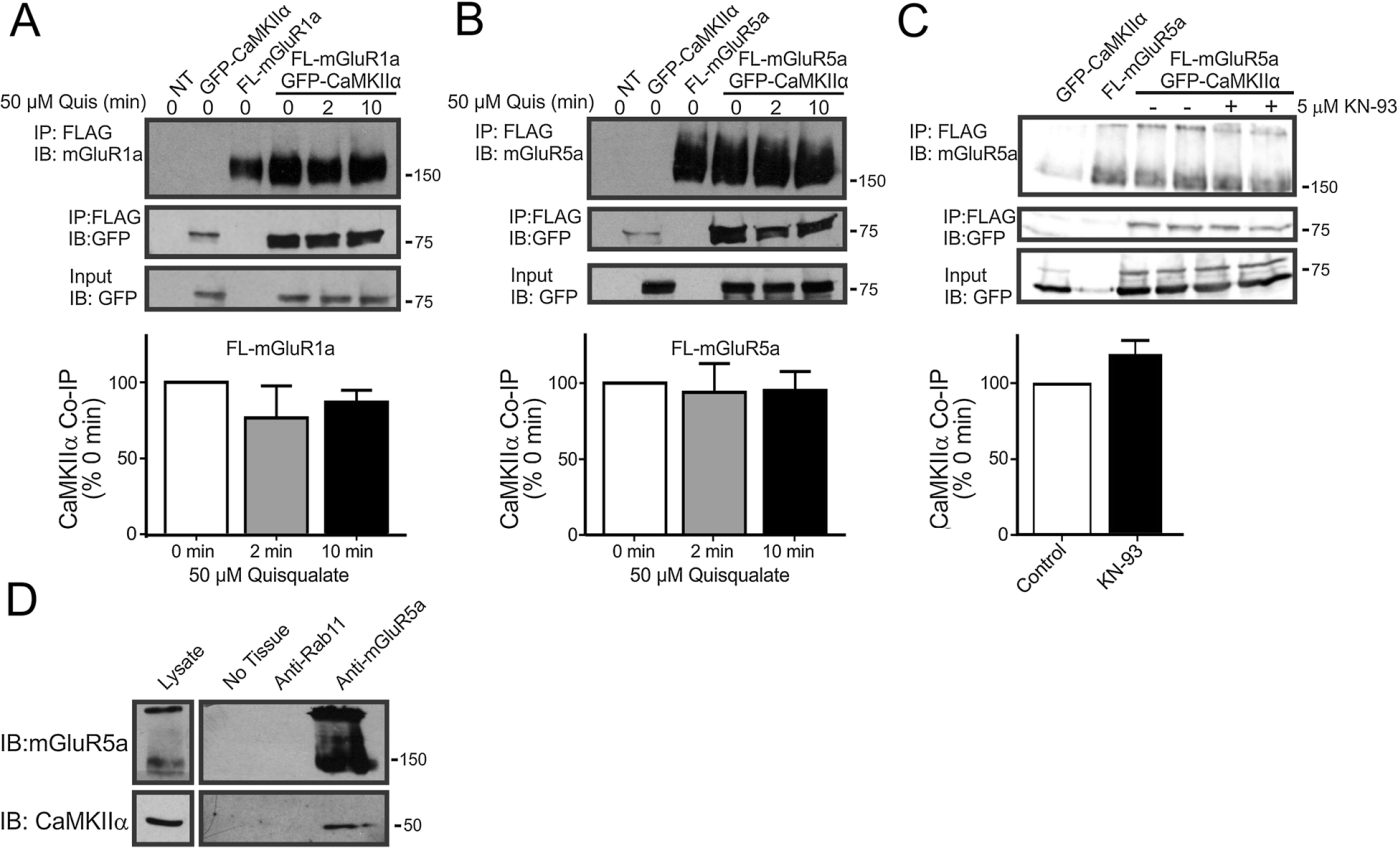
**Co-immunoprecipitation of GFP-CaMKIIα with FLAG-mGluR1a and FLAG-mGluR5a.** Representative immunoblot showing GFP-CaMKIIα co-immunoprecipitation with either **A)** FL-mGluR1a or **B)** FL-mGluR5a from HEK 293 cells transiently transfected as labeled with 3 μg of pcDNA3.1 of either FL-mGluR1a or FL-mGluR5a along with either 0.5 μg of plasmid cDNA encoding either pEGFP or GFP-CaMKIIα and treated with 50 μM quisqualate for the times indicated in the *Figure*. Bar graphs show the quantitative densitometric analysis of GFP-CaMKIIα co-immunoprecipitated with either FL-mGluR1a or FL-mGluR5a. The data represents the mean ± SD of 6 independent experiments. **C)** Representative immunoblot showing GFP-CaMKIIα co-immunoprecipitation with FLAG-mGluR5a in HEK 293 cells treated with or without 5 μM KN-93 that were transiently transfected with 2 μg of FL-mGluR5 along with 0.5 μg of GFP-CaMKIIα. Bar graphs show the quantitative densitometric analysis of GFP-CaMKIIα co-immunoprecipitated with FL-mGluR5a in the absence and following pretreatment with 5 μM KN-93 for 1 h. The data represents the mean ± SD of 6 independent experiments. **D)** Shown is a representative immunoblot of endogenous CaMKII co-immunoprecipitated with endogenous mGluR5. 1 mg of adult CD-1 mouse hippocampal tissue lysate was incubated with protein G sepharose beads and either polyclonal rabbit anti-Rab11 or anti-mGluR5 antibody. Shown is a representative immunoblot from 4 independent experiments.

To validate the potential interaction of CaMKIIα with Group I mGluRs, HEK 293 cells were transfected with either FL-epitope-tagged mGluR1a or mGluR5a along with either GFP or GFP-CaMKIIα. We found that GFP-CaMKIIα was co-immunoprecipitated with both FL-mGluR1a and FL-mGluR5a in an agonist-independent manner (Figures 1A and B). The co-immunoprecipitation of GFP-CaMKIIα with FL-mGluR1a was not dependent upon CaMKIIα catalytic activity, as the pretreatment of HEK 293 cells with 5 μM KN-93 did not affect the extent of GFP-CaMKIIα co-immunoprecipitated with the receptor (Figure 1C). Endogenous CaMKIIα could be selectively co-immunoprecipitated with endogenous mGluR5a using a rabbit polyclonal mGluR5a antibody, but not Rab11 antibody, from hippocampal mouse brain lysates (Figure 1D).

### Purified mGluR1/5 IL2 loop GST-fusion proteins interact GFP-CaMKIIα

**Figure 2:**
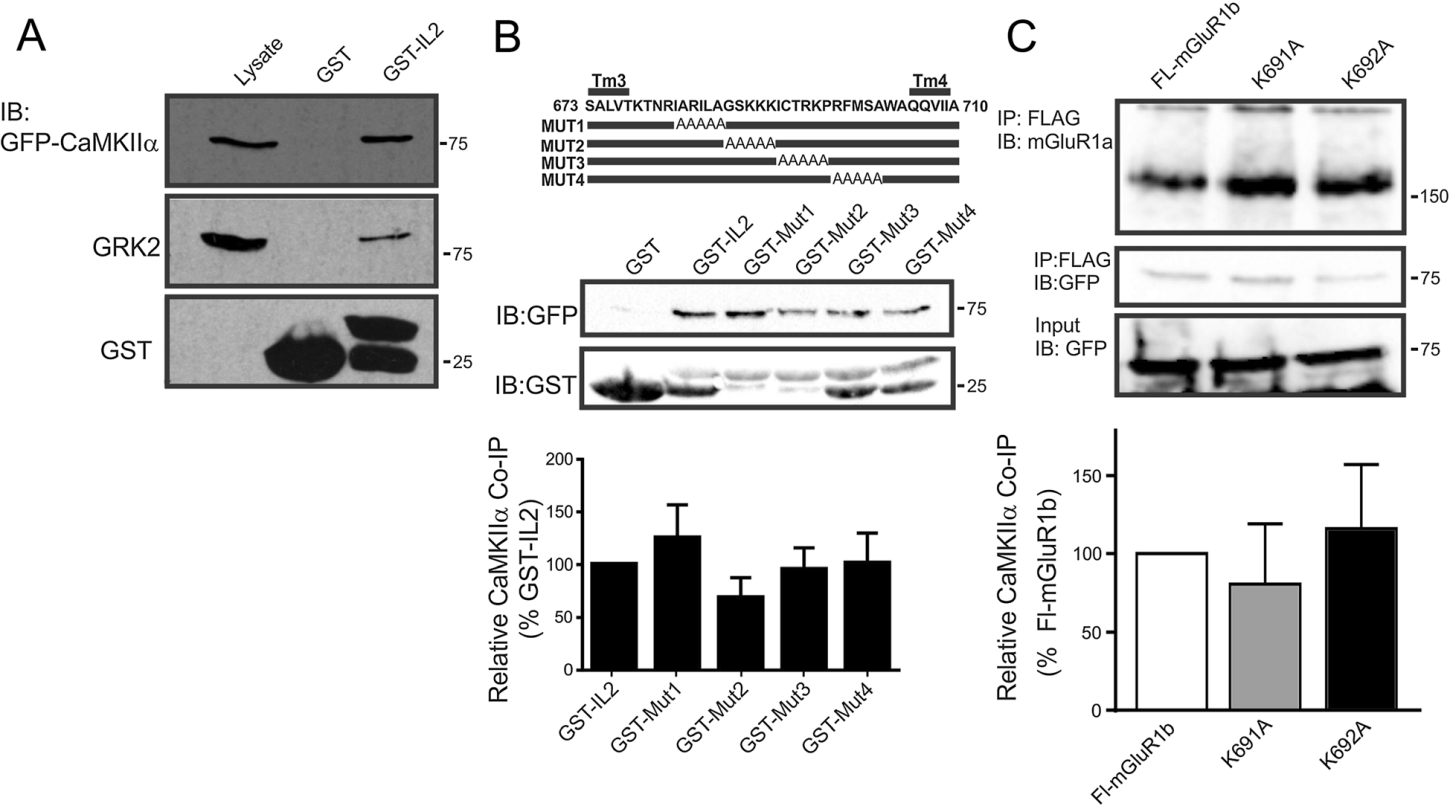
**Purified GST-fusion proteins encoding the mGluR1/5 IL2 loop domain interact with GFP-CaMKIIα. A)** Shown is a representative immunoblot showing the co-immunoprecipitation of G protein-coupled receptor kinase 2 and GFP-CaMKIIα with a GST mGluR1/5 IL2 domain fusion protein. 1 μg of GST protein was incubated with 500 μg of HEK293 cell lysates over-expressing either GRK2 or GFP-CaMKIIα. The immunoblots are representative of 3 independent experiments. **B)** Upper panel, shows the schematic representation of GST-IL2 fusion protein alanine scanning mutants previously used to identify the IL2 residues required for GRK2 binding to Group I mGluRs [39]. Middle panel shows a representative immunoblot showing the co-immunoprecipitation of GFP-CaMKIIα with the GST-IL2 fusion protein alanine scanning mutants and the expression of the GST fusions. Lower panel shows the densitometric analysis of the relative co-immunoprecipitation of GFP-CaMKIIα with the GST-IL2 fusion protein alanine scanning mutants compared to the wild-type IL2 GST fusion protein. The data represents the mean ± SD of 3 independent experiments. **C)** Representative immunoblots showing GFP-CaMKIIα co-immunoprecipitated FL-mGluR1b and FL-mGluR1b mutants (K691A and K692A) that do not bind GRK2. Also shown are FL-mGluR1a immunoprecipitates and GFP-CaMKIIα expression in cell lysates. Bar graph shows the relative co-immunoprecipitation of GFP-CaMKIIα with the FL-mGluR1b and FL-mGluR1b mutants (K691A and K692A)**.** Data shown represents the means ± SD of three independent experiments.

CaMKIIα interactions with the mGluR1/5 IL2 were identified by mass spectroscopy following the incubation of neurons with a cell permeant mGluR1/5 IL2 peptide. To confirm this interaction we purified GST and GST-IL2 from bacteria and assessed the ability of these constructs to co-precipitate either GFP-CaMKIIα or GRK2 (positive control) from HEK 293 cell lysates [39]. We found that GST-IL2 effectively co-precipitated both GFP-CaMKIIα and GRK2 (Figure 2A). We then utilized a series of GST-mGluR1/5 IL2 alanine scanning mutants that we previously employed to identify IL2 residues required for GRK2 binding in an attempt to further delineate specific mGluR1/5 IL2 residues required for GFP-CaMKIIα (Figure 2B) [39]. However, we were unable to discern discrete IL2 residues required for GFP-CaMKIIα binding (Figure 2B). Moreover, FL-mGluR1b, that lacks an extended carboxyl-terminal tail and two mGluR1b mutants defective in GRK2 binding (K691A and K692A), was still effectively able to co-immunoprecipitate GFP-CaMKIIα (Figure 2C). Thus, although CaMKIIα interacts with the IL2 of mGluR1/5, there appeared to be no specific IL2 residues that are essential for CaMKIIα binding.

### CaMKIIα contributes to agonist-stimulated endocytosis of both mGluR1a and mGluR5

**Figure 3:**
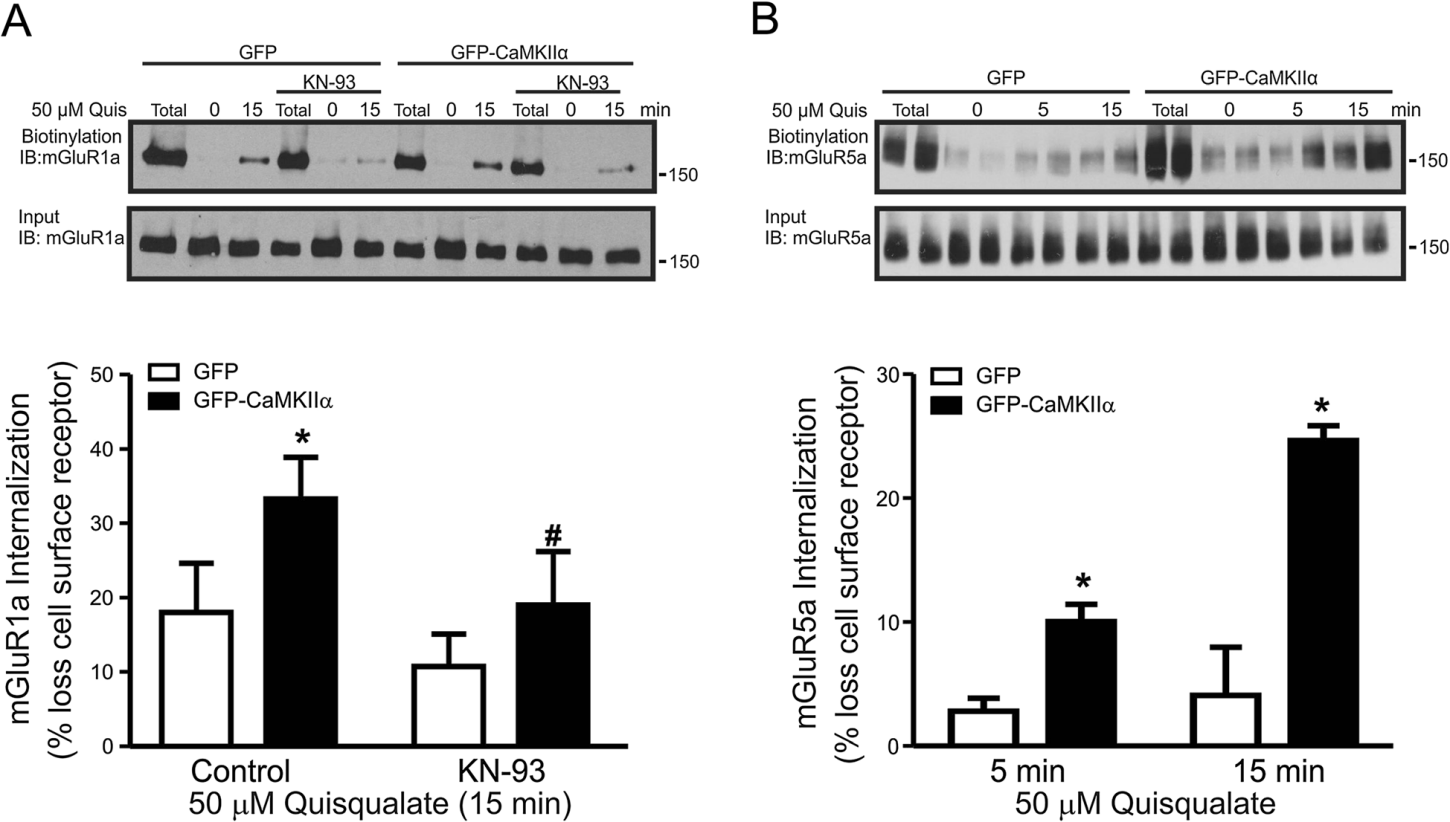
**CaMKII**α **overexpression increases agonist-stimulated mGluR1a and mGluR5 endocytosis. A)** *Upper blot* shows a representative immunoblot for cell surface biotin-labeled mGluR1a in HEK 293 cells transfected with 3 μg of pcDNA3.1 encoding FL-mGluR1a along with 0.5 μg of plasmid cDNA encoding either GFP or GFP-CaMKIIα following 50 μM quisqualate treatment following 1 h pre-treatment in the presence and absence of KN-93. *Lower blot* shows the total cell lysates (50 μg) for mGluR1a. The bar graph shows the densitometric analysis of biotin-labeled cell surface mGluR1a immunoblots normalized to total mGluR1a biotinylation. Data represents the mean ± SD of 7 independent experiments. *P < 0.05 versus GFP transfected cells. **B)** *Upper blot* shows a representative immunoblot for cell surface biotin-labeled mGluR5a in HEK 293 cells transfected with 3 μg of pcDNA3.1 encoding FL-mGluR5a along with 0.5 μg of plasmid cDNA encoding either GFP or GFP-CaMKIIα following 50 μM quisqualate treatment. *Lower blot* shows the total cell lysates (50 μg) for mGluR5a. The bar graph shows the densitometric analysis of biotin-labeled cell surface mGluR1a immunoblots normalized to total mGluR5a biotinylation. Data represents the mean ± S.E.M. of 4 independent experiments completed in duplicate. *P < 0.05 versus GFP transfected cells, #P < 0.05 versus GFP CaMKIIα control.

It has previously been reported that the antagonism of CaMKII activity can negatively regulate the internalization of mGluR1a and its alternatively spliced variants [34]. Therefore, we examined whether the overexpression of CaMKIIα would increase the agonist-stimulated endocytosis of either FL-mGluR1a or FL-mGluR5a. We found that the over-expression of GFP-CaMKIIα promoted the agonist-stimulated endocytosis of both FL-mGluR1a and FL-mGluR5a (Figures 3A and B). In agreement with the previous work of Mundell et al., 2002, the CaMKII antagonist KN-93 blocked the CaMKIIα-dependent increase of FL-mGluR1a endocytosis in GFP-CaMKIIα over-expressing cells [34] (Figure 3A). Thus, it appears that CaMKIIα contributes to the endocytosis of mGluR1a in a kinase activity-dependent manner.

### Effect of CaMKIIα over-expression on mGluR1a/5a-stimulated ERK1/2 activation

**Figure 4:**
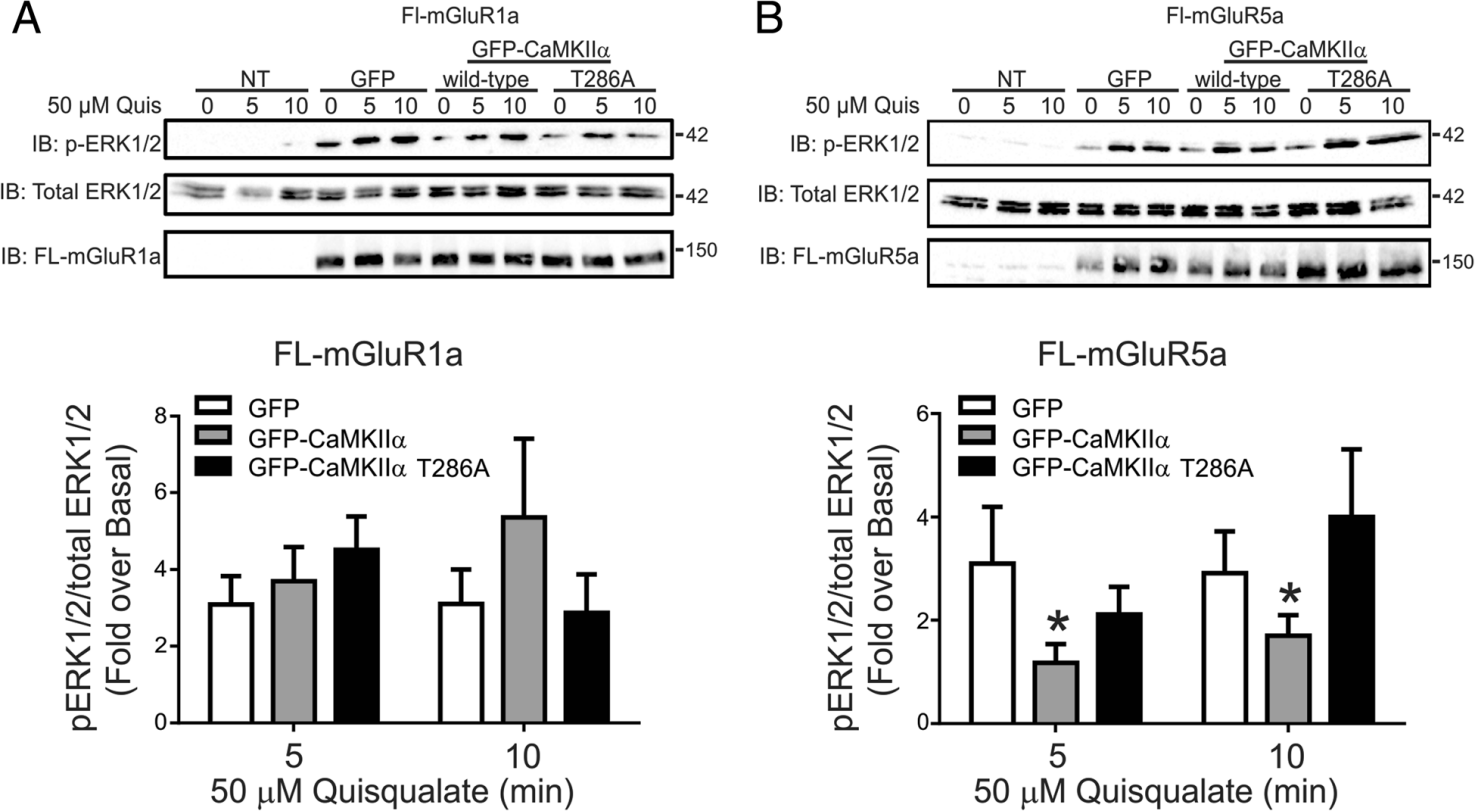
**Effect of CaMKIIα overexpression on mGluR1a- and mGluR5a- -stimulated ERK1/2 phosphorylation. A)** Shown are representative immunoblots for FL-mGluR1a expression, p-ERK1/2 activity and total-ERK1/2 expression in HEK 293 cells transiently transfected with 3 μg of pcDNA3.1 encoding FLAG-mGluR1a along with 0.5 μg of plasmid cDNA encoding either GFP, GFP-CaMKIIα or GFP-CaMKIIα-T286A in response to 50 μM quisqualate treatment for 0, 5 and 10 min. Bar graph shows the densitometric analysis of ERK1/2 phosphorylation normalized to both basal and total ERK1/2 protein expression. Data represents the mean ± SD of five independent experiments. *P < 0.05 versus GFP transfected control cells. **B)** Shown are representative immunoblots for, FL-mGluR5a expression, p-ERK1/2 activity and total-ERK1/2 expression in HEK 293 cells transiently transfected with 3 μg of pcDNA3.1 encoding, FL-mGluR5a along with 0.5 μg of plasmid cDNA encoding either GFP, GFP-CaMKIIα or GFP-CaMKIIα-T286A in response to 50 μM quisqualate treatment for 0, 5 and 10 min. Bar graph shows the densitometric analysis of ERK1/2 phosphorylation normalized to both basal total ERK1/2 protein expression. Data represents the mean ± SD of five independent experiments. *P < 0.05 versus GFP transfected control cells.

Group I mGluRs couple to the activation of ERK1/2 phosphorylation via a number of different mechanisms, that include Gα_q/11_-mediated activation of Ca^2+^ signaling leading to the activation of both protein kinase C and Src, as well as their interaction with both Pyk2 and Homer [22,40]. Therefore, we examined whether over-expression of either CaMKIIα or an autophosphorylation incompetent CaMKIIα-T286A mutant altered either FL-mGluR1a- or FL-mGluR5a-stimulated ERK1/2 phosphorylation. We found that neither GFP-CaMKIIα nor CaMKIIα-T286A over-expression affected FL-mGluR1a-stimulated ERK1/2 phosphorylation in response to 50 μM quisqualate stimulation for either 5 or 10 min (Figure 4A). In contrast, CaMKIIα over-expression significantly attenuated FL-mGluR5a stimulated ERK1/2 phosphorylation in response to 5 and 10 min treatment with 50 μM quisqualate, whereas the expression of CaMKIIα-T286A mutant had no effect on quisqualate stimulated ERK1/2 phosphorylation in FL-mGluR5a expressing cells (Figure 4B).

### Effect of Aβ42 oligomer treatment on mGluR5a CaMKIIα interactions and ERK1/2 signaling

**Figure 5:**
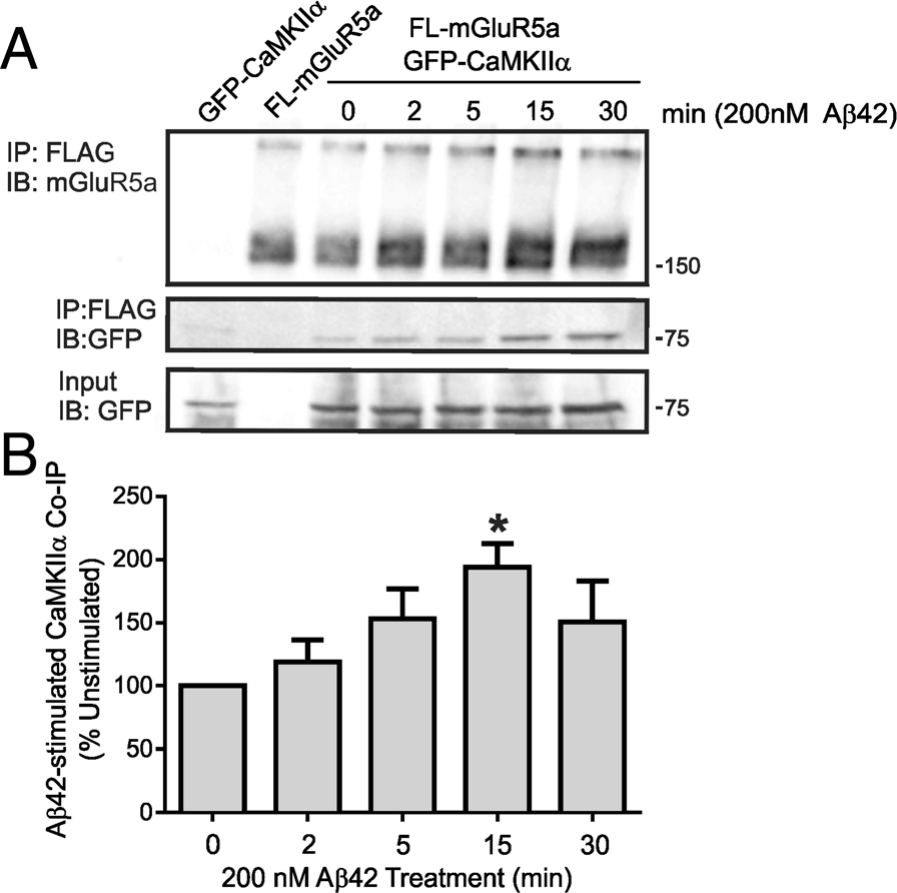
**Aβ42 oligomers increase CaMKIIα co-immunoprecipitation with mGluR5a. A)** HEK 293 cells were transiently transfected with 2 μg of pcDNA3.1 encoding FLAG-mGluR5a and 0.5 μg of plasmid cDNA encoding GFP-CaMKIIα. HEK 293 cells were treated with 200 nM of Aβ42 oligomer for 0, 2, 5, 15 and 30 min. **B)** The bar graph shows the densitometric analysis of the relative co-immunoprecipitation of GFP-CaMKIIα with FL-mGluR5a following Aβ42 oligomer treatment normalized to total mGluR5a protein expression. Data represents the mean ± SD of 5 independent experiments. *p < 0.05 versus untreated cells.

**Figure 6:**
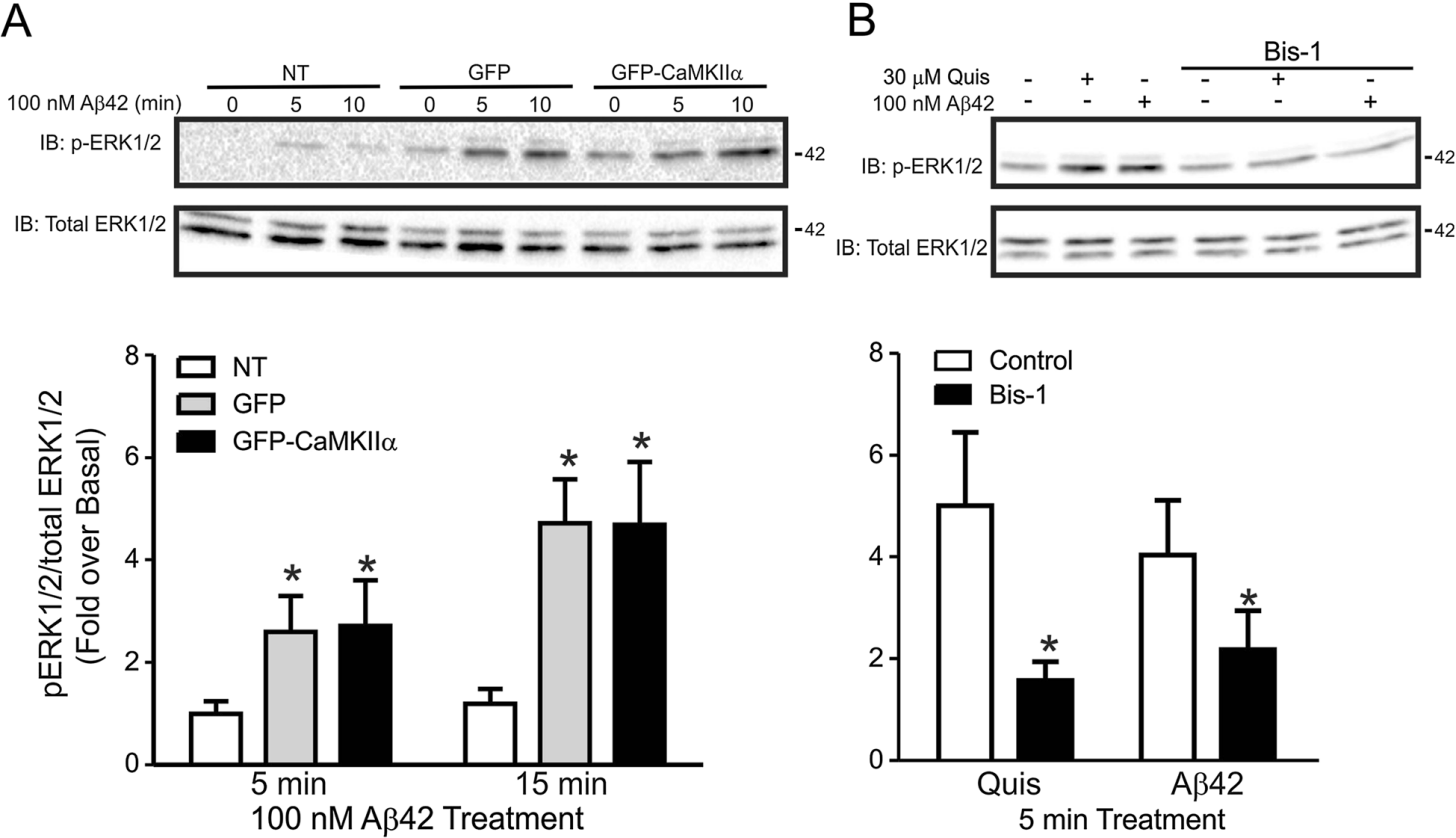
**Aβ42 oligomers stimulate ERK1/2 phosphorylation in an mGluR5a and PKC dependent manner that is independent of CaMKIIα overexpression. A)** Shown are representative immunoblots of p-ERK1/2 activity and total ERK1/2 expression in HEK 293 cells transfected with 2 μg of pcDNA3.1 encoding FLAG-mGluR5a along with either 0.5 μg of plasmid cDNA encoding either GFP or CaMKIIα and treated with 100 nM Aβ42 oligomer for 5 and 10 min. Bar graph shows the densitometric analysis of ERK1/2 phosphorylation normalized to both basal and total ERK1/2 protein expression. Data represents the mean ± SD of 4 independent experiments. *P < 0.05 versus non-transfected (NT) cells. **B)** Shown are representative immunoblots of p-ERK1/2 activity and total ERK1/2 expression in HEK 293 cells transfected with 2 μg of pcDNA3.1 encoding FLAG-mGluR5a and treated with either 50 μM quisqualate or 100 nM Aβ42 oligomer in either the presence or absence of 1 μM Bis-1. Bar graph shows the densitometric analysis of ERK1/2 phosphorylation normalized to both basal and total ERK1/2 protein expression. Data represents the mean ± SD of 7 independent experiments. *P < 0.05 versus non-transfected (NT) cells.

Aβ42 oligomers were recently shown to signal via mGluR5a [9]. Therefore, we examined whether Aβ42 oligomer-stimulated activation of mGluR5a would alter the agonist-stimulated association of GFP-CaMKIIα with FL-mGluR5a. We found that 200 nM Aβ42 oligomer treatment of HEK 293 cells transfected with FL-mGluR5a and GFP-CaMKIIα resulted in a significant time-dependent increase in GFP-CaMKIIα co-immunoprecipitated with FL-mGluR5a (Figure 5), a response we did not observe in response to receptor agonist stimulation (Figures 1A and B). Given that CaMKIIα negatively regulated agonist-stimulated mGluR5a-dependent ERK1/2 phosphorylation, we examined whether Aβ42 oligomer treatment of mGluR5a resulted in ERK1/2 phosphorylation and whether this activation was regulated by CaMKIIα interactions. We found that treatment of FL-mGluR5a expressing cells with 200 nM Aβ42 oligomers stimulated a significant increase in ERK1/2 phosphorylation, but that Aβ42 oligomer-stimulated ERK1/2 phosphorylation via the activation of FL-mGluR5a was not affected by CaMKIIα overexpression (Figure 6A). Similar to quisqualate-stimulated mGluR5a activation of ERK1/2 phosphorylation, Aβ42 oligomer-stimulated ERK1/2 phosphorylation could be antagonized by the pretreatment of HEK 293 cells with 1 mM of the PKC inhibitor Bisindolymaleimide I (Bis I) (Figure 6B). Thus, Aβ42 oligomers could stimulate ERK1/2 phosphorylation in an mGluR5a- and PKC-dependent manner that was not regulated by CaMKIIα, despite the fact that Aβ42 oligomers increased CaMKIIα association with mGluR5a.

### Effect of PrP^C^ overexpression on the subcellular distribution of CaMKIIα

**Figure 7:**
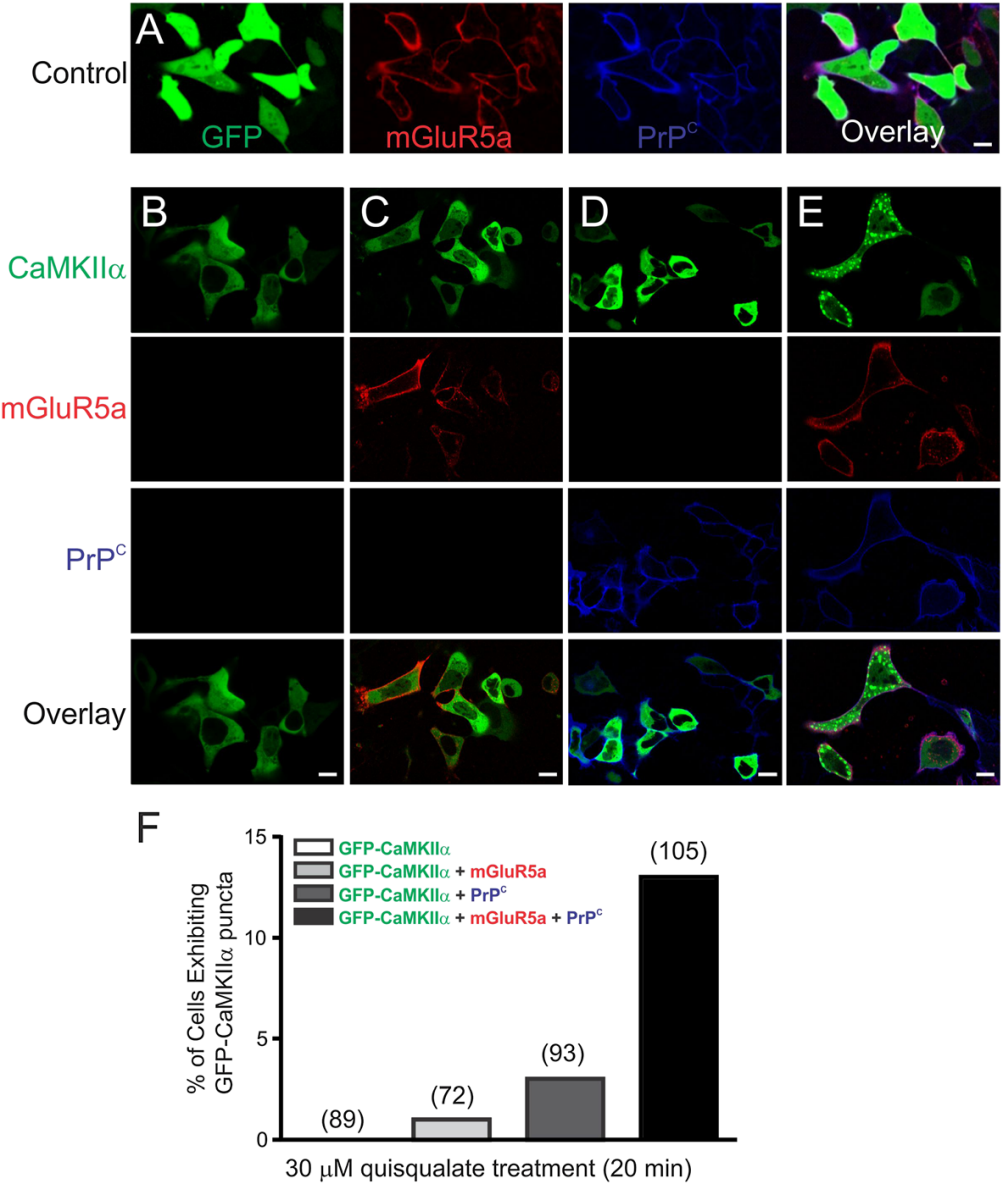
**Effect of mGluR5a and PrP** ^**C**^ **expression of GFP-CaMKII**α **subcellular localization following agonist stimulation.** HEK 293 cells were transiently transfected with different combinations of GFP-CaMKIIα (0.5 μg), FLAG-mGluR5a (2 μg), PrP^C^ (2 μg) or empty pEGFP (2 μg) pcDNA3.1 plasmid cDNA. Shown are representative confocal microscopic images of HEK 293 cells transfected with **A)** pEGFP (control) along with both FL-mGluR5a and PrP^C^, **B)** GFP-CaMKIIα alone (green), **C)** GFP-CaMKIIα along with FL-mGluR5a (red), **D)** GFP-CaMKIIα along with PrP^C^ (blue), and **E)** GFP-CaMKIIα along with both FL-mGluR5a and PrP^C^. All transfections were treated with 30 μM quisqualate for 20 min. **F)** Quantification of the number of cells exhibiting GFP-CaMKIIα puncta, number of cells imaged is shown in brackets. Data is representative 4 different experiments.

Aβ42 oligomers were previously shown to interact with PrP^C^ with high-affinity and this complex was recently shown to utilize mGluR5a as a scaffold to facilitate Aβ42 oligomer-dependent intracellular signaling [9,41,42]. Moreover, Aβ42 oligomers were shown to stimulate the subcellular redistribution of CaMKII resulting in impaired α-amino-3-hydroxy-5-methyl-4-isoxazolepropionic acid (AMPA) receptor trafficking [43]. Therefore, we assessed whether the co-expression of mGluR5a and PrP^C^ would alter the subcellular localization of GFP-CaMKIIα in HEK 293 cells. The expression of mGluR5a and PrP^C^ did not alter the subcellular distribution of GFP in HEK 293 cells (Figure 7A). In addition, 30 μM quisqualate treatment did not affect the diffuse cytosolic distribution of GFP-CaMKIIα when it was expressed alone in HEK 293 cells (Figure 7B). Co-expression of either mGluR5a or PrP^C^ alone with GFP-CaMKIIα did not alter the subcellular localization of GFP-CaMKIIα in response to 30 μM quisqualate treatment (Figures 7C and D). However, co-expression of both mGluR5a and PrP^C^ along with GFP-CaMKIIα resulted in the subcellular redistribution of GFP-CaMKIIα in intracellular cytoplasmic puncta in 13% of transfected HEK 293 cells following 30 μM quisqualate treatment (Figures 7E and F). Thus, mGluR5a and PrP^C^ were both required for agonist-stimulated alterations in the subcellular localization of GFP-CaMKIIα.

## Discussion

In the present study, we screened for novel proteins that may interact with the IL2 domain of mGluR1 and mGluR5. In doing so, we identified a number of functionally interesting putative Group I mGluR-interacting substrates, several of which were previously described as Group I mGluR-interacting proteins [19,21]. Specifically, we found that Gα_q/11_, the catalytic subunit of protein phosphatase 1γ and the regulatory subunit of protein phosphatase 2A, known mGluR5-interacting proteins, associate with the mGluR1/5 IL2 domain. Here, we show that CaMKIIα also interacted with the mGluR1/5 IL2 domain, in addition to its reported interaction with the mGluR1a and mGluR5a carboxyl-terminal tail [36,37].

CaMKIIα is known to both interact with and contribute to the desensitization of ionotropic glutamate receptors, as well as Gα_q/11_-coupled dopamine D1 and D3 receptors [30,31,44]. CaMKIIα is reported to interact with mGluR1a via its association with the mGluR1a carboxyl-terminal tail in a Ca^2+^-dependent manner and contributes to the subsequent attenuation of endogenous mGluR1a signaling in striatal neurons [36]. CaMKIIα has also been shown to interact with the carboxyl-terminal tail of mGluR5a in rat striatal neurons in a calcium dependent manner [37]. These two proteins dissociate upon activation of mGluR5 calcium and CaMKII subsequently binds to and phosphorylates the adjacent GluN2B subunit of the NMDA receptor, suggesting a mechanism by which mGluR5/CaMKIIα interactions could potentially mediate long-term potentiation [37]. We find here that CaMKIIα can be co-immunoprecipitated with both mGluR1a and mGluR5a in an agonist-independent manner and that it interacts with a GST-fusion protein encoding IL2 of mGluR1/5. This indicates that both the IL2 and carboxyl-terminal tail domains play a role in regulating CaMKIIα interactions with Group I mGluRs. The interaction of CaMKIIα with mGluR1a is reported to phosphorylate and desensitize agonist-stimulated mGluR1a signaling in striatal neurons and CaMKII activity is suggested to regulate G protein-coupled receptor kinase 2 (GRK2)-mediated desensitization of mGluR1a [35,36]. However, we previously found that DHPG-stimulated inositol phosphate signaling in mouse striatal neurons is mediated predominantly by mGluR5a [45]. Thus, either rat striatal neurons express mGluR1a at different levels than those found in mice or the interaction of CaMKII with mGluR5a in rat striatal neurons may also contribute the regulation of mGluR5a in these cultures. This desensitization may also involve CaMKII-mediated regulation of GRK2-dependent Group I mGluR desensitization, as endogenous GRK2 expression is essential for regulating mGluR5 signaling in mouse striatal neurons and both CaMKII and GRK2 bind to the mGluR1/5 IL2 domain [35,39,45].

Previous studies have demonstrated that many regulatory proteins interact with both the IL2 and carboxyl-terminal tail domains of mGluR1/5, including heterotrimeric G protein, GRK2, phospholipase D1, RalA, Arf6 and Pyk2 [3,17,22,39]. Thus, it is not unprecedented that binding to the intracellular face of an integral membrane protein would involve multiple intracellular receptor domains. However, although desirable, it is difficult, if not impossible to distinguish the relative importance of CaMKIIα interactions with the IL2 or carboxyl-terminal tail domains of mGluR1/5. Furthermore, previous studies identified interactions between the IL3 of the D3 dopamine receptor and CaMKII and the carboxyl-terminal tail of mGluR1a and CaMKII, indicating that both loop and tail domains of GPCRs may contribute to CaMKII interactions [27,30]. However, both of these studies failed to examine whether other intracellular domains of the receptor could also interact with CaMKII and rather only examined *in vitro* interactions with GST fusion proteins, which may have led to the potential misconception that CaMKII binds to a single discrete domain in both receptors.

We show that CaMKIIα overexpression increases the agonist-stimulated internalization of both mGluR1a and mGluR5a and that this is dependent upon the catalytic activity of the enzyme as KN-93 treatment blocked CaMKIIα-dependent enhancement of mGluR1a endocytosis. These results are similar to those previously reported for the GABA_B_ receptor, another Class C receptor [46]. The antagonism of CaMKIIα also reduces both the heterologous and agonist-stimulated internalization of mGluR1a and its alternative splice variants [34,35]. Interestingly, the agonist-stimulated internalization of mGluR1a appears to be impaired by its extended carboxyl-terminal tail, as the mGluR1b and mGluR1c splice variants exhibit enhanced internalization, which is effectively blocked by KN-93 [34]. In contrast, both we and Mundell and colleagues (2002) find that internalization of mGluR1a in the absence of CaMKIIα overexpression in HEK 293 cells is not effectively blocked by KN-93 treatment. Thus, although the C-terminus of mGluR1a contains the consensus site for CaMKIIα phosphorylation, the extended C-terminus may impede interactions with IL2, which we find is also involved in the interaction of CaMKIIα with the receptor. This impediment may be overcome in neurons where CaMKIIα is an abundantly expressed protein.

We find that the overexpression of CaMKIIα differentially effects the regulation of ERK1/2 phosphorylation in response to the activation of both mGluR1a and mGluR5a. Specifically, mGluR1a-stimulated ERK1/2 phosphorylation is unaffected by the overexpression of wild-type CaMKIIα. In contrast, over-expression of wild-type CaMKIIα results in the attenuation of ERK1/2 phosphorylation in response to agonist stimulation of mGluR5a. Consistent with these observations, CaMKII is known to affect DHPG-mediated ERK1/2 phosphorylation in striatal neurons [47]. Specifically, the treatment of striatal neurons with the CAMKII inhibitor, KN-62, resulted in attenuation of DHPG-induced ERK1/2 phosphorylation in the rat striatum [47]. We also find that the attenuation of mGluR5a-mediated ERK1/2 phosphorylation by CaMKIIα is dependent upon CaMKIIα activity, as the overexpression of the autophosphorylation-incompetent CaMKIIα-T286A mutant did not alter mGluR5a-stimulated ERK1/2 phosphorylation. CaMKIIα has been previously shown to phosphorylate the carboxyl-terminal tail at a CaMKII consensus sequence R/K-x-x-S/T (Thr871) localized within the carboxyl-terminal tail of mGluR1a [36]. This consensus site is also conserved within the carboxyl-terminal tail of mGluR5a (Ser860). mGluR1a also contains three additional putative consensus sites for CaMKII phosphorylation and is phosphorylated at a secondary site by CaMKII at Thr945, which is not conserved in mGluR5a [36]. The mGluR5a carboxyl-terminal tail encodes an additional seven putative CaMKII phosphorylation consensus sequences that are not conserved in mGluR1a (Ser853, Ser879, Ser871, Ser984, Ser1016, Thr1156 and Thr1165). Thus, the differential phosphorylation-dependent regulation of mGluR1a- and mGluR5a-mediated ERK1/2 phosphorylation may be the consequence of mGluR5a phosphorylation at sites not present in the mGluR1a C-terminal tail.

We and others show that CaMKII can be co-immunoprecipitated with full length mGluR1a/5a and GST fusion proteins encoding the IL2 and carboxyl-terminal tail domains of mGluR1a/5a. However, this does not necessarily indicate that CaMKII forms a stable complex with the receptors required to phosphorylate and desensitize the receptors or regulate their endocytosis and activation of ERK1/2 phosphorylation [27]. It is entirely possible that CaMKII is activated in response to Group I mGluR-stimulated Ca^2+^ release downstream of G protein activation as opposed to forming a constitutive component of the mGluR1a/5a signaling complex. Nevertheless, regardless of whether the regulation of Group I mGluR desensitization, endocytosis and ERK1/2 phosphorylation requires a stable physical interaction with the receptor, transient receptor interactions and/or the phosphorylation of downstream regulatory proteins, CaMKII clearly plays an important role in regulating Group I mGluR activity.

Recently, it has been reported that mGluR5a functions as the co-receptor for PrP^C^ and functions as a scaffold/co-receptor for both PrP^C^- and Aβ42 oligomer-mediated intracellular signaling [42,48]. Aβ42 oligomer binding to mGluR5 stimulates the clustering of mGluR5 and increases Ca^2+^ signaling [48]. We find here that Aβ42 oligomer treatment of HEK 293 cells increases the association of CaMKIIα with mGluR5a and activates mGluR5a-dependent ERK1/2 signaling to an extent that is similar to agonist stimulation. Despite the fact that Aβ42 oligomer treatment leads to increased CaMKIIα association with mGluR5a, unlike what we observe for agonist-stimulated ERK1/2 phosphorylation, CaMKIIα interactions do not antagonize Aβ42-stimulated ERK1/2 phosphorylation. Nevertheless, both agonist- and Aβ42- stimulated ERK1/2 phosphorylation are protein kinase C-dependent suggesting a role for Ca^2+^ signaling. Thus, although both mGluR5a agonists activate ERK1/2 phosphorylation via a convergent downstream mediator, quisqualate and Aβ42 stabilize mGluR5a activation states that appear to be differentially regulated by CaMKIIα.

Aβ42 oligomers have previously been demonstrated to activate both p38 MAPK and ERK1/2 via their association with the α7 nicotinic acetylcholine receptor [49]. Furthermore, Aβ42 oligomer-mediated activation of the β_2_-adrenergic receptor results in the β-arrestin-dependent activation of ERK1/2 and genetic deletion of the β_2_-adrenergic receptor ameliorates the pathophysiological deficits associated with the APPswe/PS1ΔE9 mouse model of AD [50]. In addition, Aβ42 oligomers induce alterations in the subcellular localization of CaMKIIα leading to altered AMPA receptor trafficking [44]. We find that the co-expression of both PrP^C^, an Aβ42 oligomer binding partner and mGluR5a, the co-receptor for both Aβ42 oligomers and PrP^C^ [9], results in the redistribution of GFP-CaMKIIα into intracellular cytoplasmic puncta. The mechanisms regulating CaMKII clustering in HEK 293 cells and neurons is mediated by similar mechanisms [51]. Thus, this observed change in CaMKIIα distribution in neurons leading to altered AMPA receptor localization in response to Aβ42 oligomer treatment of neurons may involve the association of Aβ42 oligomers and PrP^C^ with mGluR5a.

## Conclusions

In summary, we confirm that mGluR5a acts as a binding partner for CaMKIIα and demonstrate that in addition to associating with the mGluR5a C-terminal tail, CaMKIIα interacts with the IL2 domain of mGluR5a and mGluR1a [36]. Moreover, we find that CaMKIIα overexpression facilitates the endocytosis of both mGluR1a and mGluR5a in a manner that is dependent upon the catalytic activity of the enzyme. In contrast, CaMKIIα selectively regulates agonist-stimulated mGluR5a-mediated ERK1/2 phosphorylation, but not Aβ42 oligomer-mediated ERK1/2 phosphorylation. Moreover, CaMKIIα subcellular localization is altered by mGluR5a and PrP^C^ expression. Taken together these data suggest that not only does CaMKIIα regulate mGluR5a activity, but that Aβ42 oligomer signaling via its association with mGluR5a and PrP^C^ may contribute to pathophysiological alterations in mGluR5a cell signaling associated CaMKIIα.

## Materials and methods

### Materials

Adult CD-1 mice were from Charles River (Wilmington, MA). Human Embryonic Kidney (HEK293) Cells were from American Type Culture Collection (Manassas, VA). Cell culture reagents were from Invitrogen (Burlington, ON): Minimal Essential Media (MEM), Dulbecco’s Modified Eagle Medium (DMEM), Fetal Bovine Serum (FBS) and 0.25% Trypsin-EDTA. OmniPur Bovine Serum Albumin (BSA) was from VWR (Mississauga, ON). L-quisqualic acid (quisqualate) and KN-93 were from Tocris Bioscience (Minneapolis, MN). Biotinylation reagents EZ-Link Sulfo-NHS-SS-Biotin and NeutrAvidin Agarose Resin, as well as Pierce ECL Western Blotting Substrate and SuperSignal West Dura Chemiluminescent Substrate were purchased from Thermo Scientific (Rockford, IL). Myo-[^3^H] Inositol was purchased from Perkin Elmer (Waltham, MA). Protein G Sepharose beads were from GE Healthcare (Oakville, ON). ANTI-FLAG M2 Affinity Gel was purchased from Sigma-Aldrich (St. Louis, MO). A DC Protein Assay Kit was purchased from BioRad Laboratories (Mississauga, ON). Kodak X-Omat Blue Film was from Fisher Scientific (Ottawa, ON). Aβ42 peptide was purchased from American Peptide (Vista, CA, USA). Bisindolymaleimide I (Bis-1) hydrochloride was purchased from Calbiochem (San Diego CA, USA). CaMKII (pan), CaMKIIα, Phospho-p44/42 MAPK, p44/42 MAPK were purchased from Cell Signaling Technology (Danvers, MA). Rabbit mGluR1a and mGluR5a antibodies were purchased from Millipore (Billerica, MA), GFP antibody was purchased from Invitrogen (Mississauga, ON) and secondary mouse and rabbit antibodies were purchased from GE Healthcare (Oakville, ON) and BioRad (Mississauga, ON), respectively. GFP-CaMKIIα construct was from Dr. Paul De Koninck (Laval University, PQ).

### Cell culturing and transfection

Human embryonic kidney (HEK) 293 cells were cultured in MEM with 8% FBS. Cells were plated on 100 mm dishes and transfected using a modified calcium phosphate method [52] with cDNA amounts indicated in the *Figure Legends*. For transfection, cDNA was diluted to 450 μL in sterile distilled water, 50 μL 2.5 M CaCl_2_ added, 500 μL 2X HEPES-buffered saline (0.38 M NaCl, 0.05 M HEPES, 1.5 mM Na_2_HPO_4_, pH 7.05) added drop-wise and mixed gently before transfection mixture was added to cells. Cells were washed 16–20 hours post transfection and then allowed to recover in new media before experimentation. For co-immunoprecipitation, cells recovered for 24 hours. For all other experiments, cells recovered for 6–8 hours and then were reseeded into 6-well dishes and allowed to recover for 18 hours.

### Mass spectroscopy identification of mGluR1/5 interacting proteins

Neuronal cultures were prepared from cortical and striatal brain tissue from CD-1 E14.5 mouse embryo brains. Animal procedures were approved by The University of Western Ontario Animal Care Committee. After dissection, the tissue was submitted to trypsin digestion followed by cell dissociation using a fire-polished pasteur pipette. Cells were plated on poly-L-ornithine coated dishes in neurobasal media supplemented with N2 and B27 supplements, 2 mM of glutamax, 50 μg/ml penicillin, and 50 μg/ml streptomycin. Cells were incubated at 37°C and 5% CO2 in a humidified incubator and cultured for 12 days *in vitro* (DIV) with media replenishment every 4 days as previously described [45]. Neurons (1 x 10^7^ cells) seeded in each of three 100 mm dishes were incubated for 60 min with a 20 μM final concentration of a Tat-mGluR1/5-FLAG peptide (Ac-YGRKKRRQRRRIARILAGSKKKICTRKPRFMSDYKDDDDK-NH2) that encoded a cell permeant HIV Tat peptide at its amino-terminal end, followed by the amino acid sequence corresponding to the conserved second intracellular loop domains of mGluR1/5 and followed by a FLAG epitope tag. Subsequently, neurons were washed 3 times with PBS and solubilized in 1% Triton-X lysis buffer (25 mM HEPES, pH 7.5, 300 mM NaCl, 1.5 mM MgCl_2_, 0.2 mM EDTA and 1% Triton X-100) containing protease inhibitors (1 mM AEBSF and 20 μg/mL of both leupeptin and aprotinin). Cellular debris was precipitated by centrifugation at 13,000 g for 30 min at 4°C. Cellular lysates were pre-cleared by incubation with a 20 μL volume of Protein A agarose beads for 6 h. Lysates were then incubated with a 40 μL volume of FLAG M2 resin for 4 h and washed 3X with Lysis buffer and 3 times with 50 mM NH_4_HCO_3_ pH 7.8. Subsequently, co-immunoprecipitates were eluted with 500 mM NH_4_OH at pH 11 in three 100 μL volumes and lyophilized to remove NH_4_OH. The samples were then resuspended in a 100 μL volume of H_2_O and subjected to a second round of lyophilization. Subsequently, samples were resuspended in a 50 μL volume of NH_4_HCO_3_ pH 8.0 and directly digested with sequencing-grade trypsin (Promega). The resulting peptide mixture was then analyzed by liquid chromatography-tandem mass spectrometry using a LTQ-XL Linear Ion Trap Mass Spectrometer (Thermo Scientific). The acquired tandem mass spectra were searched against a FASTA file containing the human NCBI sequences using a normalized implementation of SEQUEST running on the Sorcerer platform (Sage-N Research). The resulting peptide identifications returned by SEQUEST were filtered and assembled into protein identifications using peptide and protein prophets (Institute of Systems Biology, Seattle) as described previously [53].

### Immunoblotting

HEK 293 cells were transiently transfected with various cDNA constructs as described in the *Figure Legends*. One day after transfection, cells were starved for 1 hour in HBSS (1.2 mM KH_2_PO_4_, 5 mM NaHCO_3_, 20 mM HEPES, 11 mM Glucose, 116 mM NaCl, 4.7 mM KCl, 1.2 mM MgSO_4_, 2.5 mM CaCl_2_, pH 7.4) and subsequently stimulated as indicated in the *Figure Legends*. Cells were washed on ice with cold Phosphate-Buffered Saline (PBS: 137 mM NaCl, 2.7 mM KCl, 4.3 mM Na_2_HPO_4_, 1.4 mM KH_2_PO_4_, pH 7.2) and then lysed on a rocking platform for 15 minutes at 4°C for using 0.1% Triton-X 100 lysis buffer (0.025 M HEPES, 300 mM NaCl, 1.5 mM MgCl_2_, 0.2 mM EDTA, 0.1% Triton-X) with added protease inhibitors: 1 mM AEBSF, 10 μg/ml leupeptin, and 5 μg/ml aprotinin. Lysate was collected and centrifuged at 15,000 RPM for 15 minutes at 4°C. 250 μg of each lysate was incubated with FLAG (FL)-agarose beads (50 μl bead slurry) for 1–2 hours. Beads were washed three times with cold PBS. Samples were eluted using 3x SDS sample buffer with 2-mercaptoethanol and separated by SDS-PAGE and co-immunoprecipitated proteins were detected by Western Blot.

### Brain lysate co-immunoprecipitation

The hippocampus was removed from CD-1 Adult Mice and placed in 0.5% Triton-X 100 lysis buffer with protease inhibitors. It was homogenized using a polytron and solubilized for 2 hours at 4°C. Lysate was then centrifuged at 15,000 RPM for 15 minutes at 4°C and 1 mg of protein was incubated with protein G-sepharose with or without mGluR5 antibody (Millipore, 1.5 μg) to immunoprecipitate mGluR5. Samples were eluted using 3x SDS sample buffer with 2-mercaptoethanol and separated by SDS-PAGE. Membranes were immunoblotted for immunoprecipitated mGluR5 (Millipore, 1:1000) and co-immunoprecipitated CaMKIIα (Santa Cruz, 1:250).

### GST pull down

GST-mGluR1a-IL2 and mutants were cloned into a pGEX4T1 vector and transformed into *E.coli* recombinant bacteria [39]. *E. coli* bacteria were grown at 37°C with shaking until OD_600_ was 0.6-1.0. Cultures were then induced with 1 mM isopropyl 1-thio-β-D-galactopyranoside for 3 hours at 23°C. Cells were pelleted and lysed in lysis buffer (500 mM NaCl, 0.5% NP-40, 50 mM Tris pH7.6, 5 mM EDTA, 5 mM EGTA) containing protease inhibitors (2 mM AEBSF, 50 mg/ml aprotinin, 20 mg/ml leupeptin) and sonicated (3 times for 10 seconds) at 4°C. Insoluble material was pelleted at 15000 g for 15 minutes at 4°C. 50 μl of Glutathione-Sepharose bead slurry was incubated overnight with 1 ml of solubilized protein to purify GST-fusion constructs. Glutathione-sepharose beads were then washed 3 times in PBS and 500 μg of HEK 293 cell lysates overexpressing GFP-CaMKIIα was added to the GST-fusion peptide bound to matrix and rotated for 1 hour at 4°C. Glutathione-sepharose beads were then washed 6 times in PBS and eluted with 3X SDS loading buffer containing β-mercaptoethanol. Samples were subjected to SDS-PAGE and membranes were immunoblotted with GFP to determine if GFP-CaMKIIα was pulled down with the GST-mGluR1a-IL2 peptides as described previously [39].

### ERK activation

Twenty-four hours after transfection, cells were reseeded into 6-well dishes. The following day, cells were starved in DMEM overnight. On the day of experiment, cells were starved for an additional hour in HBSS. Cells were then stimulated at 37°C with either 50 μM quisqualate or 100 nM Aβ42 oligomers as indicated in the *Figure Legends*. For experiments using the PKC inhibitor, 1 μM Bis-1 was added 30 minutes prior to stimulating cells. Cells were subsequently lysed in lysis buffer (25 mM HEPES, 300 mM NaCl, 1.5 mM MgCl_2_, 200 μM EDTA, 0.1% Triton-X) containing protease and phosphatase inhibitors (1 mM AEBSF, 25 mg/ml aprotinin, 10 mg/ml leupeptin, 10 mM NaF, 100 μM Protein concentration was determined using a Bradford protein assay. The lysates were mixed with SDS loading buffer containing β-mercaptoethanol prior to gel loading. ERK1/2 phosphorylation was determined by immunoblotting for phospho-ERK1/2 and total ERK1/2 and the ratio was normalized to basal levels.

### Biotinylation internalization assay

HEK293 cells were transiently transfected with receptor (FL-mGluR1a and FL-mGluR5a) and either pEGFP (control) or GFP-CaMKIIα (3 μg of receptor and 0.5 μg of GFP constructs). Cells were serum starved for 1 hour in HBSS for at 37°C on the morning of the experiment. Cells were washed and incubated on ice for 20 minutes in HBSS. Plasma membrane proteins were biotinylated at 4°C with EZ-Link Sulfo-NHS-SS-Biotin in HBSS and then incubated at 4°C in 100 mM glycine in HBSS for 30 minutes to quench biotinylation. Cells were then stimulated with 50 μM quisqualate for 0, 5 and 15 minutes, which allowed the receptor to internalize. Remaining cell surface biotin was stripped using 100 mM sodium 2-mercaptoethanesulfonate (MesNa) in TE Buffer (150 mM NaCl, 1 mM EDTA, 20 mM Tris, pH 8.6) with 0.2% BSA. A control without stimulation or stripping was kept on ice and used to assess amount of total cell surface receptor. Cells were lysed, biotinylated protein pulled down with NeutrAvidin agarose resin (50 μL bead slurry), eluted with 3x SDS sample buffer containing 2-Mercaptoethanol, separated by SDS-PAGE and immunoblotted for mGluR1a and mGluR5a (Millipore, 1:1000). Internalization of the receptor at various time points with and without GFP-CaMKIIα was compared to GFP transfected control cells. Results are expressed as percent cell surface internalization. Protocol was modified slightly for mGluR1a experiments. Following serum starving, cells were pretreated for 1 hour with or without 1.0 μM KN-93 in HBSS. For stimulation, cells were stimulated with 50 μM quisqualate for 0 and 15 minutes.

### Aβ42 oligomer preparation

Lyophilized Aβ42 peptides stored at -80°C were allowed to equilibrate to room temperature prior to dilution to 1 mM with 1,1,1,3,3,3-hexafluoro-2-propanol (HFIP). HFIP was evaporated in a vacuum centrifuge in order to form Aβ42 peptide films and films were then stored at -80°C. Prior to use, Aβ42 peptide films were diluted in dimethylsulphoxide (DMSO) to 1 mM and sonicated for 10 minutes in a Branson sonicator. Aβ42 peptides were then subsequently diluted to 100 μM in ice-cold F-12 cell culture media (phenol free red), vortexed immediately for 30 seconds, and incubated at 4°C for 24 hours in order to form Aβ42 oligomers.

### Confocal microscopy

Confocal microscopy was performed using a Zeiss LSM-510 laser scanning microscope equipped with a Zeiss 63X 1.4 numerical aperture oil immersion lens. Live cell imaging was performed on HEK293 cells in 35 mm glass-bottomed plates. mGluR5a was labeled with rabbit anti-FLAG conjugated Zenon Alexa Fluor 647 antibody and PrP^C^ was labeled with mouse anti-PrP^C^ conjugated Zenon Alexa Fluor 555 IgG2B antibody. Visualization of labelled proteins with GFP-CaMKIIα was performed by triple excitation (488/543/647 nm), emission band pass from 505–530 (GFP), long pass at 560 (Alexa Fluor 555) and 660 (Alexa Fluor 647) filter sets. For internalization experiments, mGluR5a was labelled with rabbit anti-FLAG-conjugated Zenon Alexa Fluor 555 antibody. Visualization of antibody-labelled receptor with GFP-CaMKIIα was performed by dual excitation (488/543 nm) and emission band pass from 505–530 (GFP) and long pass at 560 (Alexa Fluor 555) filter sets. Receptor was stimulated with the addition of 30 μM quisqualate (final concentration) for 20 minutes.

### Statistical analysis

Immunoblots were quantified using Image Lab software. GraphPad Prism software was used to analyze data for statistical significance as well as to analyze and fit dose–response curves. Statistical significance was determined by either an unpaired two-tailed *t*-test or by one-way ANOVA followed by Tukey’s post hoc multiple comparison’s test.

## Abbreviations

Aβ42: β42-amyloid
AD: Alzheimer’s disease
AMPA: α-amino-3-hydroxy-5-methyl-4-isoxazolepropionic acid
Bis-1: Bisindolymaleimide I
CaMKII: Ca^2+^/calmodulin kinase II
ERK1/2: extracellular-regulated protein kinase 1/2
GPCR: G protein-coupled receptor
GRK2: G protein-coupled receptor kinase 2
HEK 293: Human embryonic kidney
IL2: Intracellular loop domain 2
mGluR: Metabotropic glutamate receptor
PrP^c^: Cellular prion protein
quisqualate: L-quisqualic acid
